# Comparison of health-related quality of life in children and adolescents with monosymptomatic nocturnal enuresis under therapy versus allergic bronchial asthma, diabetes mellitus type I, and juvenile idiopathic arthritis – a KINDL-R-based study

**DOI:** 10.1007/s00467-025-07042-3

**Published:** 2025-11-19

**Authors:** Marcus O. Klein, Nora Duncker, Alexander Thews, Michael Kalab, Sebastian Schulz-Jürgensen

**Affiliations:** 1 Department of Pediatrics I, University Hospital of Schleswig-Holstein – Campus Kiel, Arnold-Heller-Straße 3, Haus C, 24105 Kiel, Germany; 2 Department of Geriatrics, Marienhospital, Hamburg, Germany; 3 Department of Anesthesia, Israelite Hospital, Hamburg, Germany; 4 Institute for Medical Informatics and Statistics, Christian-Albrechts-University of Kiel, Kiel, Germany; 5 Department of Pediatrics, University Hospital Hamburg-Eppendorf, Hamburg, Germany

**Keywords:** KINDL-R, MNE, Children, HRQoL, Chronic diseases, Comparison

## Abstract

**Background:**

Monosymptomatic nocturnal enuresis (MNE) affects 10–15% of children at school age. Influences on psychological well-being and self-confidence were repeatedly described, but effects on health-related quality of life (HRQoL) have been rarely reported. The aim of this study is to examine the HRQoL under therapy by using KINDL-R and to compare it with three recognized chronic diseases.

**Methods:**

Questionnaires were sent to all patients with MNE of the special outpatient clinic for enuresis (age 7–17 years; groups: I: 7–13, II: 14–17 years; at least 3 months of therapy, no achieved dryness). Simultaneously, patients from special outpatient clinics for allergic bronchial asthma (ABA), diabetes mellitus type I (DMI), and juvenile idiopathic arthritis (JIA) were asked to take part in the study.

**Results:**

Included patients: 47 MNE (I:41/II:6)/59 ABA (I:34/II:25)/57 DMI (I:31/II:26)/37 JIA (I:18/II:19). Patient reports showed no significant differences between the cohorts in both age groups examined with regard to the total score and six individual dimensions. In the additional “chronic-generic” module, patients with MNE in the age of 7–13 years showed significantly lower values than all other study cohorts (*p* < 0.001).

**Conclusions:**

Patients under therapy for MNE without achieving dryness showed no significant differences in overall HRQoL or individual dimensions compared to the reference groups, but a significantly lower HRQoL in the chronic-generic module in children and parents (group 7–13 years) and in adolescents (14–17 years of age). This result is consistent with reported limitations in self-esteem and HRQoL before therapy and supports the need and importance of adequate therapy for MNE.

**Graphical abstract:**

**Figure:**
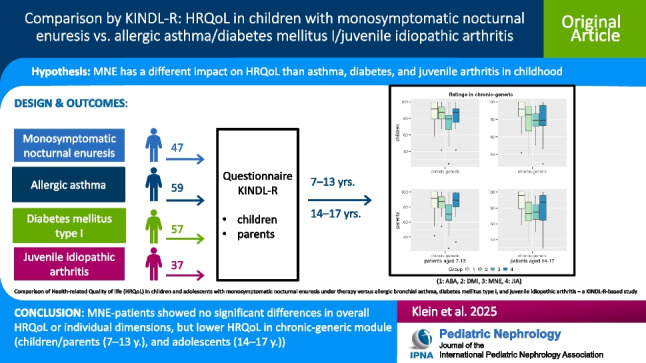
A higher-resolution version of the Graphical abstract is available as  Supplementary information

**Supplementary Information:**

The online version contains supplementary material available at 10.1007/s00467-025-07042-3.

## Introduction

Monosymptomatic nocturnal enuresis (MNE) is one of the most common chronic diseases in childhood, with a prevalence of 7–15% in the 7-year-old age group and around 5% of 10-year-olds [1], with boys being affected approximately twice as often as girls [1, 2]. A spontaneous healing tendency of approximately 10–15% per year is described [2, 3], but patients in adolescence and young adulthood are still affected [1]. The pathophysiology is assumed to have a multifactorial genesis including deep sleep with arousal disorder, deficiency of antidiuretic hormone (ADH) with polyuria, and bladder dysfunction with hyperactive bladder [4].

MNE is repeatedly described as putting a burden on children [5], particularly psychological effects [6, 7] and effects on self-confidence [8–11]. Only in recent years have individual reports been published on the impact on the health-related quality of life (HRQoL) of those affected [12–14]. A Turkish study reported a significantly lower HRQoL measured with PedsQL questionnaires in untreated patients with enuresis compared to a healthy comparison group [12]. A Japanese study using the Kid-KINDL questionnaires showed reduced HRQoL before and improvement after treatment [13]. Both studies had an additional focus on maternal HRQoL measured with WHOQOL-BREF and SF-36 [12, 13]. A study from our own working group compared HRQoL from enuresis under ongoing treatment to normative data and a population-based study with the KINDL questionnaire, surprisingly showing comparable overall HRQoL compared to the general population [14]. However, a comparison of MNE patients with patients with other common chronic diseases has not yet been investigated.

To be able to compare the measured HRQoL scores, patients from other specialized outpatient departments were analyzed for comparison. The diagnoses of allergic bronchial asthma (ABA), diabetes mellitus type I (DMI), and juvenile idiopathic arthritis (JIA) were chosen due to the availability of the respective outpatient departments and relatively well-defined diagnoses treated there.

Since the therapeutic intervention does not automatically mean nocturnal dryness, this work will specifically investigate the quality of life of patients undergoing therapy but still affected by enuresis as a basis for comparison.

## Methods

The MNE group included all patients in the age group of 7–17 years who had been treated for at least 3 months but were not yet dry and who attended the enuresis consultation at the children's clinic at the UKSH campus in Kiel in the years 2013–2015. Exclusion criteria were the presence of daytime symptoms (daytime incontinence, urge symptoms, reduced bladder volume in the voiding diary, pathologic result of sonography) as part of the work-up in the outpatient department (non-monosymptomatic enuresis). Only patients with monosymptomatic enuresis were asked to participate in the study.

The samples of the three other comparison diseases were recruited during the same period from the respective special outpatient clinics of the children’s clinic on the UKSH campus in Kiel.

The inclusion criteria for children and adolescents with DMI were the presence of diabetes-specific antibodies at manifestation, and long-term insulin therapy by subcutaneous injection (pen or pump) for at least one year.

The inclusion criteria for children and adolescents with ABA were evidence of specific sensitization, an initial one-second capacity (FEV1) < 70%, a maximum expiratory flow at 25% of the vital capacity (MEF25) < 60%, and long-term (uninterrupted) inhaled glucocorticoid therapy for at least a year.

The inclusion criteria for children and adolescents with JIA were chronic arthritis in at least one joint (exclusion of other causes), the occurrence of inflammation in the last six months, and continuous therapy during at least six months with non-steroidal anti-inflammatory drugs (NSAIDs) and steroids, methotrexate (MTX), or biologics.

Exclusion criteria in all four cohorts were other chronic diseases and severe cognitive impairments that did not allow attendance at a regular school.

The validated KINDL-R questionnaire in the version for children and parents was used to examine HRQoL [15]. The questionnaire consists of 24 statements (items), each of which should be rated with 5 possible answers (never, rarely, sometimes, often, always). From the answers, a score was calculated for the six dimensions of the test (physical well-being, emotional well-being, self-esteem, family, friends, and everyday functioning (school)) and transformed to a scale of 0–100 with higher scores indicating a better quality of life; further on, a total score can be calculated [16]. An additional “chronic-generic” module assessing the impact of prolonged disease duration on HRQoL is also available and useful to compare chronic diseases in this context. This module includes six questions regarding fear that the disease gets worse, anxiety due to the illness, ability to cope well with the illness, being treated like a baby by the parents, missing content at school, and wishing that nobody notices the illness.

The questionnaires for parents and children were sent to the families by mail, including an accompanying letter asking for independent answering and separate envelopes for return mail.

Kruskal–Wallis tests were performed to determine differences in the scores between the groups. The data were further analyzed with the Tukey test to determine the different pairs after notable Kruskal–Wallis tests. To assess differences in the age and gender distributions between the groups, Fisher’s exact test was used. All performed tests were two-sided at a significance level of 0.05. All statistical analyses were conducted using R (version 4.3.1).

This is an observational study. The Ethics Committee of the Medical Faculty of the Christian-Albrechts-University of Kiel has confirmed that no ethical consideration exists in the two studies (studies D 422/13 (MNE versus ABA), D 439/13 (JIA vs. DMI and ABA)), which we compared here*.* The group of ABA was included in both studies, but in our comparison, it was only included once. Informed consent was obtained from all individual participants included in the study and their parents.

## Results

The study included 47 children and adolescents with MNE, 59 with ABA, 57 with DMI, and 37 with JIA between 7 and 17 years of age according to the inclusion criteria. The distribution of patients across the two age groups and gender is depicted in Table 1, and the distribution of the parents involved can be seen in Table 2.

**Table 1:** Cohorts of patients (group, age, total, sex (male:female))

Group	7–13 years	14–17 years
MNE	41//31: 10 (75.6: 24.4%)	06//03: 03 (50.0: 50.0%)
ABA	34//24: 10 (70.6: 29.4%)	25//16: 09 (64.0: 36.0%)
DMI	31//16: 15 (51.6: 48.4%)	26//12: 14 (46.2: 53.8%)
JIA	18//06: 12 (33.3: 66.7%)	19//04: 15 (21.1: 78.9%)

**Table 2:** Cohorts of parents (group, age, total, sex (father:mother:both))

Group	7–13 years	14–17 years
MNE	41//08: 33: 00 (24.4: 75.6: 0%)	06//01: 05: 00 (19.5: 80.5: 0%)
ABA	34//00: 32: 02 (0: 94.1: 5.9%)	25//03: 20: 02 (12.0: 80.0: 8.0%)
DMI	31//00: 24: 07 (0: 77.4: 22.6%)	26//03:19:04 (11.5: 73.1: 15.4%)
JIA	18//02: 16: 00 (11.1: 88.9: 0%)	19//03: 16: 00 (15.8: 84.2: 0%)

There is a significant difference in the gender distribution between the groups of the study participants (7–13 years: P = 0.008, 14–17 years: *P* = 0.039), which is due to the female dominance in the JIA cohort. There was no statistically significant difference between the cohorts in the age distribution of the study participants.

In both age groups and in all cohorts, the parent-report was largely carried out by the mothers. This results in no significant difference between the groups.

**Fig. 1:**
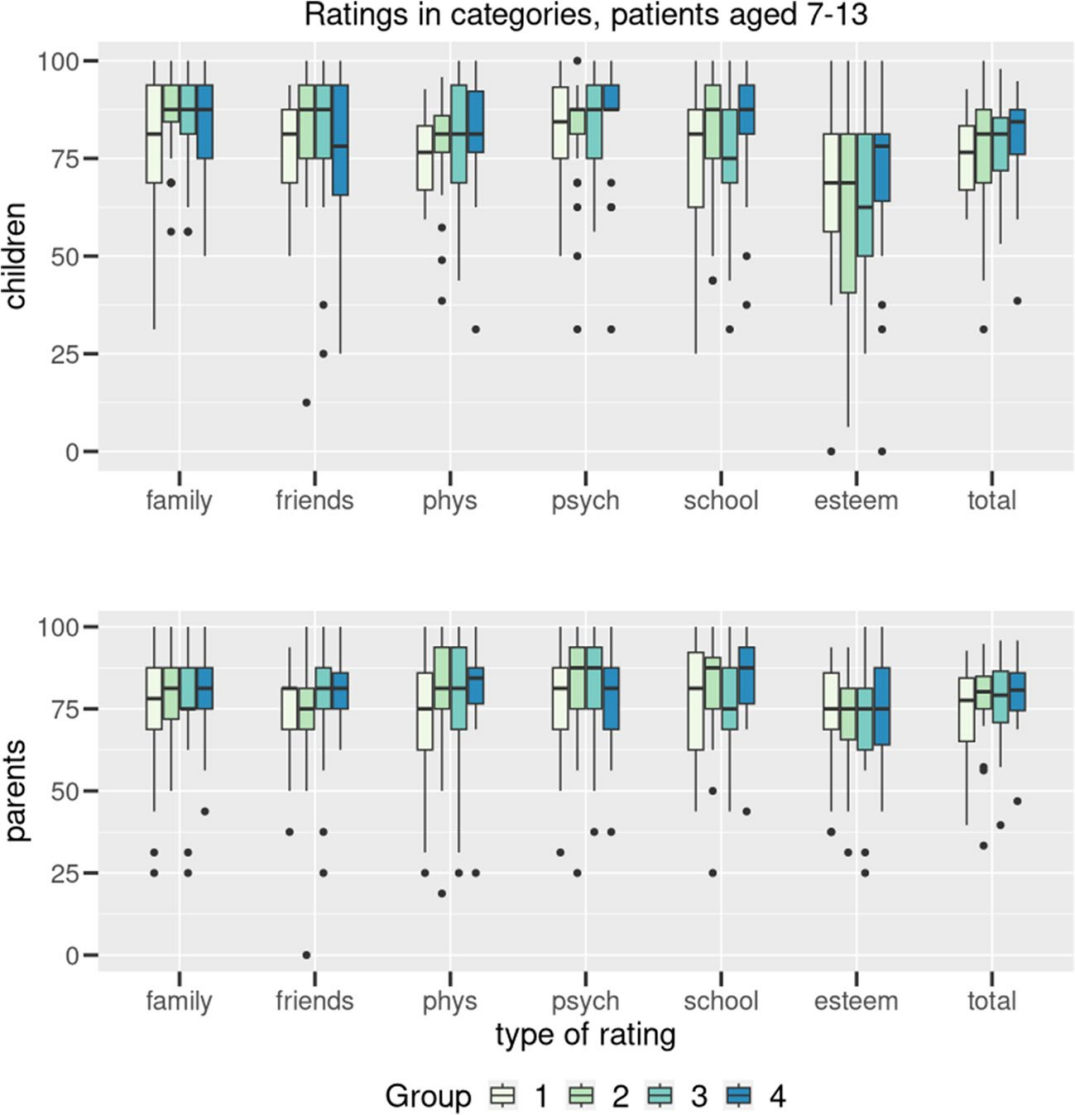
Results (group 7–13 years) transformed to 100 for the 6 dimensions (family, friends, physical wellbeing, psychological wellbeing, school, self-esteem) and total for the four conditions (1: ABA, 2: DMI, 3: MNE, 4: JIA). Boxplots indicating median, interquartile range/IQR (box), 1.5 × IQR (whiskers) and outliers (dots). Data are given for patients’ (A) and parents’ (B) reports

**Fig. 2:**
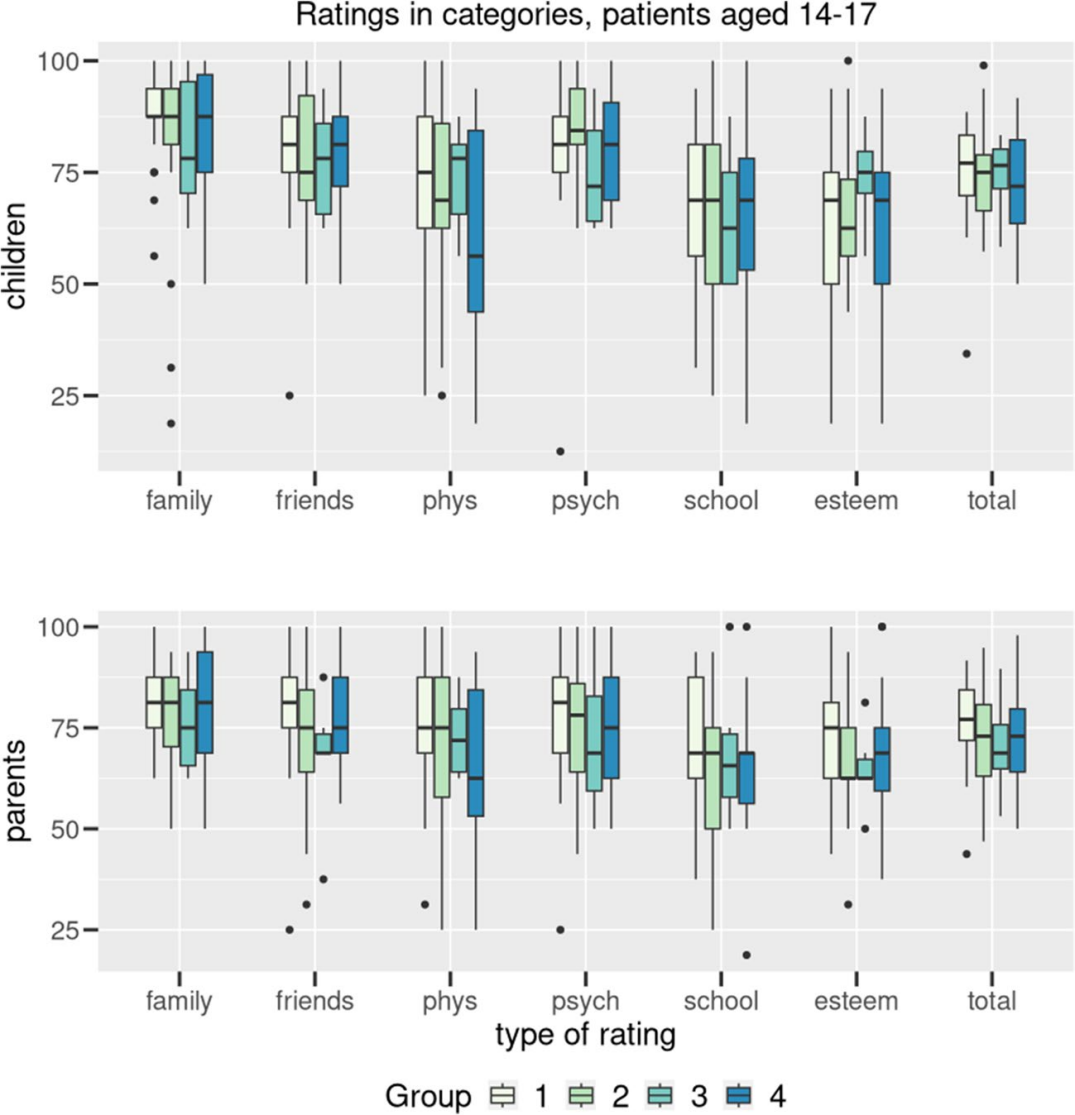
Results (group 14–17 years) transformed to 100 for the 6 dimensions (family, friends, physical wellbeing, psychological wellbeing, school, self-esteem) and total for the four conditions (1: ABA, 2: DMI, 3: MNE, 4: JIA). Boxplots indicating median, interquartile range/IQR (box), 1.5 × IQR (whiskers) and outliers (dots). Data are given for patients’ (A) and parents’ (B) reports

Figure 1 shows the scale values of the group 7–13 years in self- and parent-report for overall quality of life and individual dimensions in all four cohorts. Figure 2 shows the group of 14 to 17 years in self- and parent-report scores for total quality of life and individual dimensions in all four cohorts.

No significant difference was found between the group of children with enuresis and the groups of children with other chronic diseases in both age groups, neither in the total score nor in the individual dimensions. Only in the area of family well-being did the children 7–13 years show a non-significant (*p* = 0.06) difference compared to all three other groups.

**Fig. 3:**
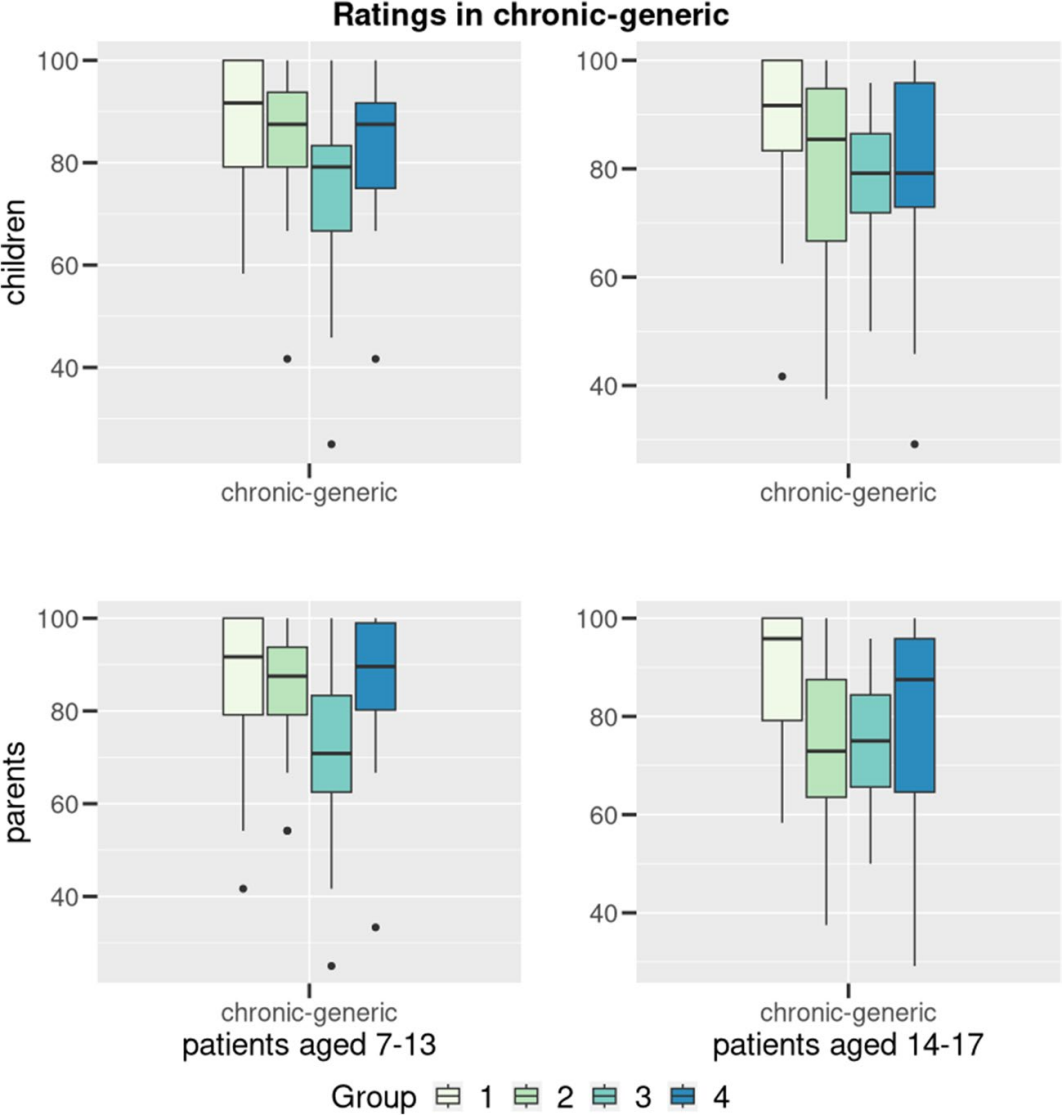
Results (group 7–13 years above, 14–17 years down) transformed to 100 for the chronic-generic dimension dimensions and total for the four conditions (1: ABA, 2: DMI, 3: MNE, 4: JIA). Boxplots indicating median, interquartile range/IQR (box), 1.5 × IQR (whiskers) and outliers (dots). Data are given for patients’ (A) and parents’ (B) reports

Figure 3 shows the scale-values transformed to 100 for the groups 7–13 and 14–17 years in self- and parent-report of the chronic-generic module in all four cohorts.

In the chronic-generic module, the children with enuresis showed a significantly lower score in the 7–13-year-olds (*p* < 0.001) than the three other groups. This difference cannot be seen in the group 14–17 years.

From the parents’ perspective, there is a significantly lower score for the group 7–13 years (*p* < 0.001) and 14–17 years (*p* = 0.003) compared to all three other groups concerning the chronic-generic module.

## Discussion

### Comparison of overall HRQoL between diseases

The total HRQoL score shows no statistically significant difference between children with enuresis, asthma, diabetes, and rheuma undergoing therapy. The same applies to the six individual dimensions. It can therefore be said that children and adolescents who suffer from MNE experience a comparable reduction in their quality of life as those with ABA, DMI, and JIA. This finding can be derived from both self- and parent-report and is also independent of the age group investigated.

There is a significant difference in the gender distribution between the groups (7–13 years: *p* = 0.008, 14–17 years: *p* = 0.039), which is in favour of girls due to the distribution in the JIA cohort. Various studies have shown that girls, regardless of age, usually report a significantly lower quality of life than boys of the same age [17–21]. This was also shown for the group of children with JIA [17, 22]. However, others have found no gender-specific differences [23]. Therefore, in the case of a different gender distribution, statistically significant differences between children with enuresis and rheuma could be possible.

It remains undisputed that MNE per se can represent a high burden not only for the children and adolescents affected [7], but also for the parents, although mothers were primarily examined in this regard [12, 13, 24]. Further study results underline that parents’ sensitive approach to the disease is not a given, but can be crucial. It was shown that mothers and fathers can be exposed to excessive demands, which significantly influence the success of therapy [25].

Another study describes that parents only seek medical help when the quality of life is so impaired by nighttime enuresis that it affects other areas of life. In addition, there is often no awareness that there are generally effective therapeutic options for treating enuresis [26]. The significantly worse values in the chronic-generic module show the relevance of the effects of the symptoms.

### Special considerations

Enuresis nocturna is a disease whose medical significance may be underestimated due to the absence of daytime symptoms, bodily complaints or pain, the spontaneous remission rate, and the intimacy of symptoms. In addition to the widespread opinion of a strong psychiatric or psychological component and a social taboo surrounding enuresis, a lack of pressure to treat it can be recognized because the consequences mentioned in terms of long-term effects arise gradually and may not be associated with enuresis at all when brought into context. The results collected here should lead to greater attention to MNE in its importance as a chronic disease. Destigmatization could be the first step in offering affected children and adolescents access to therapy, which should be quick and effective to avoid long-term consequences of the disease. In addition, an adequate and direct comparison of the untreated children with enuresis with a healthy comparison group could then be carried out. In the clinical classification by the International Children’s Continence Society (ICCS), MNE is defined as one form of incontinence [27]. However, the ICD10 classifies MNE among the psychiatric diagnoses (F98.0), possibly adding to the social taboo of the disease.

### Importance of chronicity

It has also been shown that mothers of affected children or adolescents understand that wetting is involuntary. However, the older the children get, the lower their tolerance to wetting becomes [28].

It should be kept in mind that all of the studies mentioned and cited in this article come from different cultural backgrounds and can therefore only be combined to a limited extent in their statements. Furthermore, the “chronic-generic” additional module of the KINDL-R shows that children with enuresis describe their well-being as significantly worse in this area. This may indicate that they are particularly concerned about the continued persistence of nighttime enuresis, i.e., chronicity, and that this is putting them under emotional strain. The questions on this dimension show the importance of this individual dimension, as it includes not only strong feelings such as “fear” and “sadness” but also the components of the personal “comfort zone” and “growing up”. The low scores of children with enuresis in this dimension in particular indicate insecurity and may be due to the intimacy of the symptom as well as the feeling that enuresis is a step away from “being taken seriously” and the meaning of the introductory quote with regard to child development: “The child who is anxious or has a low self-esteem because of the wetting is best treated by making him/her dry!” [29]. It is also conceivable that the poorer scores in this individual dimension are due to a lack of recognition of the disease or rather a lack of acknowledgment as a disease. Asthma, diabetes, and rheumatism are more socially accepted than nocturnal enuresis and may therefore be perceived differently, including by those affected.

Nevertheless, further studies from the Asian region confirm the result of chronicity being a special concern in enuresis found in this study. A work from Taiwan indicates that older children and those who are characterized by more severe symptoms report increased difficulties when asked about their psychological and emotional state [30]. A Chinese study states that enuresis is associated with an increased risk of behavioral problems, emotional instability, and academic deficits [31]. In this context, another study from China should be mentioned that investigated adult patients with enuresis in more detail and found “serious social and psychological effects” if the symptoms persisted into adult age [32].

### Parents’ role and parents’ view

The results of the parent surveys cannot be statistically compared with the information provided by the children themselves, because it is an external vs. internal view. Nevertheless, it is helpful to compare the reports by description to gain a different perspective.

Parent- and self-reports are very close to each other on most dimensions. However, the deviations in the dimensions of self-esteem and family are striking. This survey suggests that parents rate their children’s self-esteem higher than they do themselves. In the studies on self-confidence cited above [8–10], parent-reports were not examined.

In both age groups and in all cohorts, the parent-report was largely carried out by the mothers. Although this does not result in a significant difference between the groups, an influence on the result per se could be discussed.

### Scientific background

Dealing with MNE requires a high degree of discipline from parents, which is why children may perceive their parents as supportive and parents see themselves more as “controllers” in the family, and this is also reflected in the questionnaires. In the 14–17 age group, there is a similar perception between children and parents regarding quality of life. The assumption that different diseases of the genitourinary tract lead to a reduction in quality of life, which is comparable to other somatic diseases and in some specific areas can even be more stressful for those affected, was addressed at the same time as this work in an incontinence study from 2014. This approaches the description of the suffering by focusing on the outcome after a fixed duration of therapy. Using quality of life as a parameter, the data show that three months of incontinence therapy makes a significant difference in HRQoL [33]. Therefore, a noticeable trend points in such a direction and needs to be further investigated using a larger sample, especially of adolescents with enuresis between the ages of 14 and 17. A literature search has not revealed any dedicated studies on this topic, though.

### Limitations

Since our study was carried out in a special outpatient clinic, it is possible that more severely affected cases were included and thus influenced the above-mentioned results. Because of the monocentric analysis and the reduction of enuresis by age, the number of participants, especially in the group of 14–17 years, is very small and may not be representative. This is even more important if the groups are divided by gender.

For a more complete picture, a longitudinal examination before, during, and after therapy for MNE would be useful. In this case, the possibly different parent views of mothers and fathers should be included in this study. Furthermore, a study focused on the stressful aspects of the chronicity of enuresis should be discussed. A multicentric study should be performed to include a sufficient number of participants to validate the results.

## Conclusion

The assessment of overall HRQoL and its specific dimensions, utilizing the KINDL-R tool during ongoing treatment for nocturnal enuresis, reveals no significant differences when compared to conditions such as ABA, DMI, and JIA. This classifies the impact of nocturnal enuresis on quality of life within the spectrum of recognized chronic somatic illnesses affecting children and adolescents, despite its lack of visibility during daytime hours. However, the ratings in the chronic-generic module for the children with enuresis in self-report and parent-report for 7–13-year-olds and in parent-report for 14–17-year-olds are significantly lower than in all three comparison cohorts, indicating the special burden of chronicity in enuresis. This supports the importance of adequate and sustainable therapy for enuresis nocturna.

## Supplementary Information

Below is the link to the electronic supplementary material.

Graphical abstract (PPTX 127 KB)

## Data Availability

Raw data are available from the authors under reglementation of the European laws of data security.
