# Supplementary material for: Comparison of health-related quality of life in children and adolescents with monosymptomatic nocturnal enuresis under therapy versus allergic bronchial asthma, diabetes mellitus type I, and juvenile idiopathic arthritis – a KINDL-R-based study
Source: Pediatr Nephrol. 2025 Nov 19;41(4):1079–86. doi: 10.1007/s00467-025-07042-3 (PMC12953464; doi:10.1007/s00467-025-07042-3)
Supplement: Supplementary file 1 — Graphical abstract (PPTX 127 KB) [file 467_2025_7042_MOESM1_ESM.pptx]

## Slide 1
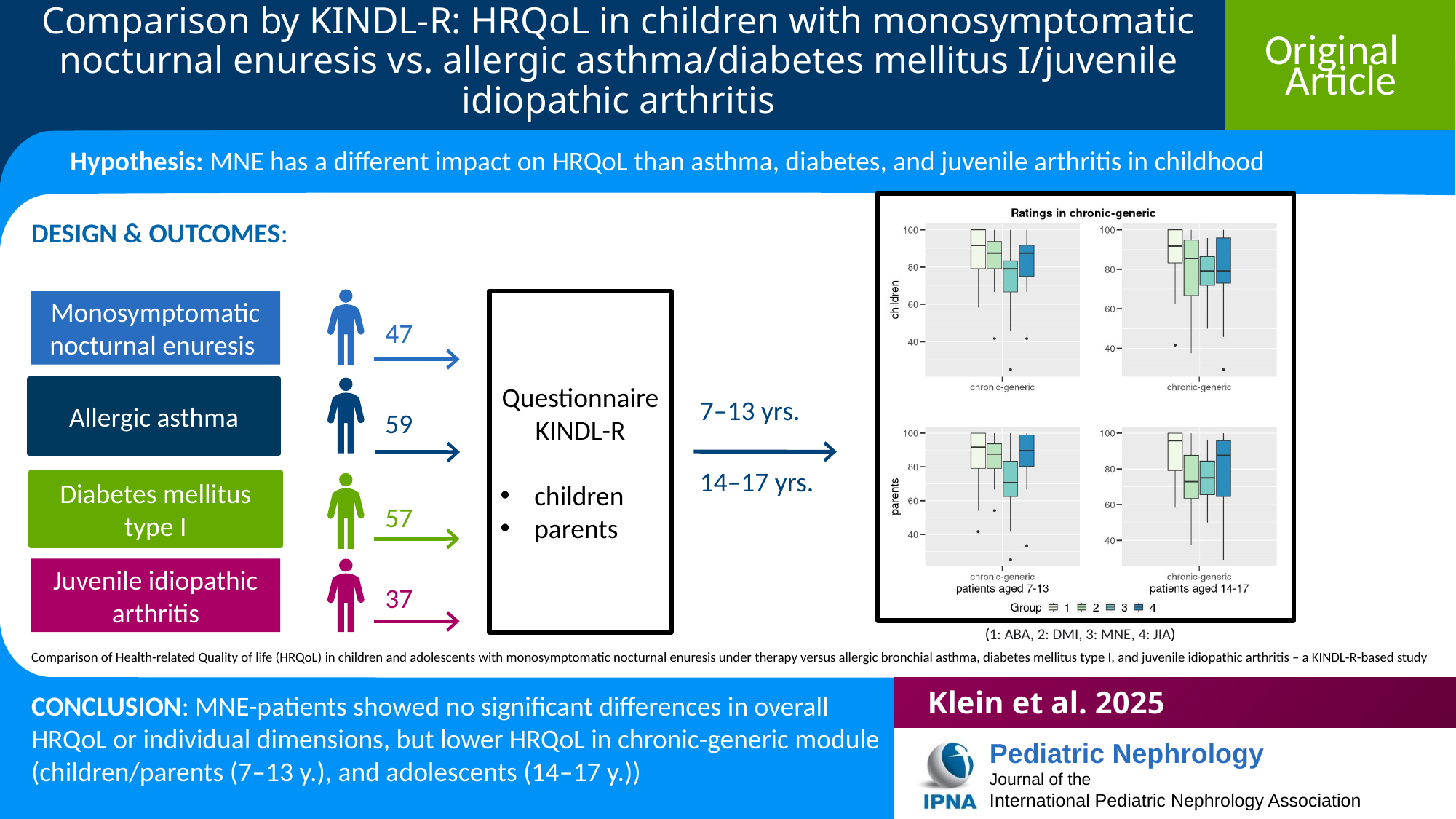

Comparison by KINDL-R: HRQoL in children with monosymptomatic nocturnal enuresis vs. allergic asthma/diabetes mellitus I/juvenile idiopathic arthritis
Hypothesis: MNE has a different impact on HRQoL than asthma, diabetes, and juvenile arthritis in childhood
DESIGN & OUTCOMES:
Monosymptomatic nocturnal enuresis
Questionnaire KINDL-R
children
parents
47
Allergic asthma
7–13 yrs.
59
14–17 yrs.
Diabetes mellitus type I
57
Juvenile idiopathic arthritis
37
(1: ABA, 2: DMI, 3: MNE, 4: JIA)
Comparison of Health-related Quality of life (HRQoL) in children and adolescents with monosymptomatic nocturnal enuresis under therapy versus allergic bronchial asthma, diabetes mellitus type I, and juvenile idiopathic arthritis – a KINDL-R-based study
Klein et al. 2025
CONCLUSION: MNE-patients showed no significant differences in overall HRQoL or individual dimensions, but lower HRQoL in chronic-generic module (children/parents (7–13 y.), and adolescents (14–17 y.))
